# CRISPR: a new principle of genome engineering linked to conceptual shifts in evolutionary biology

**DOI:** 10.1007/s10539-018-9658-7

**Published:** 2019-01-19

**Authors:** Eugene V. Koonin

**Affiliations:** National Center for Biotechnology Information, National Library of Medicine, Bethesda, MD 20894 USA

**Keywords:** CRISPR-Cas systems, Adaptive immunity, Self versus non-self discrimination, Lamarckian evolution, Horizontal gene transfer

## Abstract

The CRISPR-Cas systems of bacterial and archaeal adaptive immunity have become a household name among biologists and even the general public thanks to the unprecedented success of the new generation of genome editing tools utilizing Cas proteins. However, the fundamental biological features of CRISPR-Cas are of no lesser interest and have major impacts on our understanding of the evolution of antivirus defense, host-parasite coevolution, self versus non-self discrimination and mechanisms of adaptation. CRISPR-Cas systems present the best known case in point for Lamarckian evolution, i.e. generation of heritable, adaptive genomic changes in response to encounters with external factors, in this case, foreign nucleic acids. CRISPR-Cas systems employ multiple mechanisms of self versus non-self discrimination but, as is the case with immune systems in general, are nevertheless costly because autoimmunity cannot be eliminated completely. In addition to the autoimmunity, the fitness cost of CRISPR-Cas systems appears to be determined by their inhibitory effect on horizontal gene transfer, curtailing evolutionary innovation. Hence the dynamic evolution of CRISPR-Cas loci that are frequently lost and (re)acquired by archaea and bacteria. Another fundamental biological feature of CRISPR-Cas is its intimate connection with programmed cell death and dormancy induction in microbes. In this and, possibly, other immune systems, active immune response appears to be coupled to a different form of defense, namely, “altruistic” shutdown of cellular functions resulting in protection of neighboring cells. Finally, analysis of the evolutionary connections of Cas proteins reveals multiple contributions of mobile genetic elements (MGE) to the origin of various components of CRISPR-Cas systems, furthermore, different biological systems that function by genome manipulation appear to have evolved convergently from unrelated MGE. The shared features of adaptive defense systems and MGE, namely the ability to recognize and cleave unique sites in genomes, make them ideal candidates for genome editing and engineering tools.

## Introduction

Thanks to the unprecedented success of Cas9 endonucleases as the new generation of genome editing tools, in recent years, comparative genomics, structures, biochemical activities and biological functions of CRISPR (Clustered Regularly Interspaced Palindromic Repeats)-Cas (CRISPR-associated proteins) systems and individual Cas proteins have been explored with an intensity that is hardly matched by the study of any other class of biological entities, at least as far as microbes are concerned (Barrangou et al. 2007; Barrangou and Horvath 2017; Hille et al. 2018; Jiang and Doudna 2017; Komor et al. 2017; Mohanraju et al. 2016; Sorek et al. 2013; Wright et al. 2016). The CRISPR-Cas systems store memory of past encounters with foreign DNA in unique spacer sequences derived from viral and plasmid genomes and inserted into CRISPR arrays. Transcripts of the spacers, along with portions of the surrounding repeats, are utilized as guide CRISPR (cr)RNAs to recognize the cognate sequences in the foreign genomes and thus direct Cas nucleases to unique cleavage sites. The existence of specific, long term immune memory qualifies CRISPR-Cas as bona fide are adaptive (acquired) immune systems.

Because CRISPR-Cas are programmable immune systems that can adapt to target any sequence, they are not subject to extreme diversifying selection that led to the evolution of the immense variety of restriction-modification enzymes, the most abundant form of innate immunity in prokaryotes (Pingoud et al. 2014). Nevertheless, CRISPR-Cas systems evolve in a regime that is common to all defense system, namely continuous arms race with genetic parasites, primarily viruses, resulting in rapid evolution of at least some *cas* gene sequences (Takeuchi et al. 2012), and notable diversity of the gene compositions and genomic architectures of the CRISPR-*cas* loci, which translates into diversification of the molecular mechanisms of defense (Koonin et al. 2017a, b; Makarova et al. 2011a, b, 2015).

In this article, I address the fundamental, general biological issues that emerge through the study of the CRISPR-Cas systems. The first of these is the “Lamarckian” character of the evolutionary process engendered by CRISPR-Cas. I discuss the interplay of Lamarckian-type direct adaptation with selection and the conditions that enable this type of evolution. The second fundamental theme is the apparent coupling between the adaptive immune response and an alternative defense strategy, namely, “altruistic” programmed cell death or dormancy induction: infected cells seem to “decide” to commit suicide when immunity fails. Finally, I address the unexpected relationships between mobile genetic elements and CRISPR-Cas evolution which demonstrate the evolutionary entanglement between defense systems and those very genetic elements against which they protect the host. I generalize on this subject to formulate principles of evolution for defense and developmental systems that function via genome manipulation. What is more, the same properties of proteins encoded by MGE that make them a valuable commodity for recruitment by defense systems during evolution underlie their utility for the development of genome editing tools.

## Molecular organization and functionality of CRISPR-Cas

The CRISPR-Cas systems represent one of the nucleic acid-guided forms of defense, along with eukaryotic RNAi and prokaryotic Argonaute-based systems (Koonin 2017). Unlike the Argonaute mechanisms and most of the branches of RNAi, but similarly to the PIWI RNA systems in eukaryotes (Iwasaki et al. 2015), CRISPR-Cas mediates *bona fide* adaptive immunity. The CRISPR-*cas* genomic loci are modified to target the genome of a unique pathogen or its closest relatives with exceptional specificity and efficiency. These loci typically consist of a CRISPR array, i.e. between two and several hundred direct, often partially palindromic, exact repeats [25–35 base pairs (bp) each] that are separated by unique spacers (typically, 30–40 bp each), and the adjacent cluster of multiple *cas* genes that are organized in one or more operons. The CRISPR-Cas immune response consists of three stages: (1) adaptation, (2) expression/processing, and (3) interference. At the adaptation stage, a distinct complex of Cas proteins binds to a target DNA, migrates along that molecule and, typically after encountering a distinct, short (2–4 bp) motif known as PAM (Protospacer-Adjacent Motif), cleaves out a portion of the target DNA, the protospacer, and inserts it into the CRISPR array between two repeats (most often, at the beginning of the array) so that it becomes a spacer. Some CRISPR-Cas systems employ an alternative mechanism of adaptation, namely spacer acquisition from RNA via reverse transcription by a reverse transcriptase (RT) encoded in the CRISPR-*cas* locus. At the expression stage, the CRISPR array is transcribed into a single, long transcript, the pre-cr(CRISPR)RNA, that is processed into mature crRNAs, each consisting of a spacer and a portion of an adjacent repeat, by a distinct complex of Cas proteins or a single, large Cas protein (see below). At the final, interference stage, the crRNA that typically remains bound to the processing complex is employed as the guide to recognize the protospacer or a closely similar sequence in an invading genome of a virus or plasmid that is then cleaved and inactivated by a Cas nuclease (s). Because the CRISPR-Cas systems modify the genome content in response to an environmental cue (an invader genome) and store the memory of such encounters, allowing them to efficiently and specifically protect the host from the same or related parasites, they are often regarded as a device implementing Lamarckian-type inheritance. This brief description is an over-simplified schematic that inevitably omits many important details of CRISPR-Cas functioning. Such details can be found in many recent reviews on different aspects of CRISPR-Cas biology (Barrangou and Horvath 2017; Jackson et al. 2017; Jiang and Doudna 2017; Mohanraju et al. 2016).

At the molecular level, the CRISPR-Cas systems have a readily definable modular organization (Makarova et al. 2013a, b, 2015). The two principal parts of the CRISPR-Cas are the adaptation and effector modules that consist, respectively, of the suites of *cas* genes encoding proteins involved in spacer acquisition (adaptation) and pre-crRNA processing, followed by the target recognition and cleavage (interference). In most of the CRISPR-Cas systems, the adaptation module consists of the Cas1 and Cas2 proteins that form a complex, in which Cas1 is the endonuclease (integrase) involved in the cleavage of both the source, protospacer-containing DNA and the CRISPR array, whereas Cas2 forms the structural scaffold (Amitai and Sorek 2016). In many CRISPR-Cas variants, additional Cas proteins, such as Cas4 or Cas3 also contribute to the adaptation stage, in some of the CRISPR-Cas systems forming fusions with Cas1 or Cas2. In a sharp contrast to the relatively simple and uniform organization of the adaptation module, the effector modules are highly diverse, and their variation forms the basis of the current classification of CRISPR-Cas systems. Primarily through the comparison of the effector module architectures, all CRISPR-Cas systems are divided into Class 1, with multisubunit effector complexes comprised of several Cas proteins, and Class 2, in which the effector is a single, large, multidomain protein (Koonin et al. 2017a, b; Makarova et al. 2015). Among other distinctions, Class 1 and Class 2 CRISPR-Cas systems substantially differ in the mechanisms of pre-crRNA processing. In Class 1 systems, the crRNAs are generated by a dedicated complex of multiple Cas proteins (Charpentier et al. 2015). In Class 2 systems, processing is catalyzed either by an external bacterial enzyme, RNAse III, with the help of an additional RNA species, the trans-acting CRISPR (tracr)RNA (Chylinski et al. 2013), or by the same effector protein that is involved in the target cleavage (East-Seletsky et al. 2016; Fonfara et al. 2016). The composition and organization of the *cas* genes encoding effector module components have been further analyzed to delineate 6 types and 24 subtypes within the two CRISPR-Cas classes (Koonin et al. 2017a, b; Makarova et al. 2015). Various proteins involved in ancillary roles, such as regulation of the CRISPR response and other, still poorly characterized functions, can be provisionally assigned to a third, accessory module (Makarova et al. 2013a, b, 2014, 2015; Mohanraju et al. 2016). The modules of the CRISPR-Cas systems are partially autonomous as demonstrated by their frequent recombination and by the existence of isolated adaptation and effector modules in many bacterial and archaeal genomes (Makarova et al. 2015). However, it is important to note that the functional separation between the modules is only a rough approximation because some Cas proteins, in particular, Class 2 effectors, appear to be involved in all stages of the CRISPR response.

## The (quasi)Lamarckian character of adaptive immunity

**Fig. 1:**
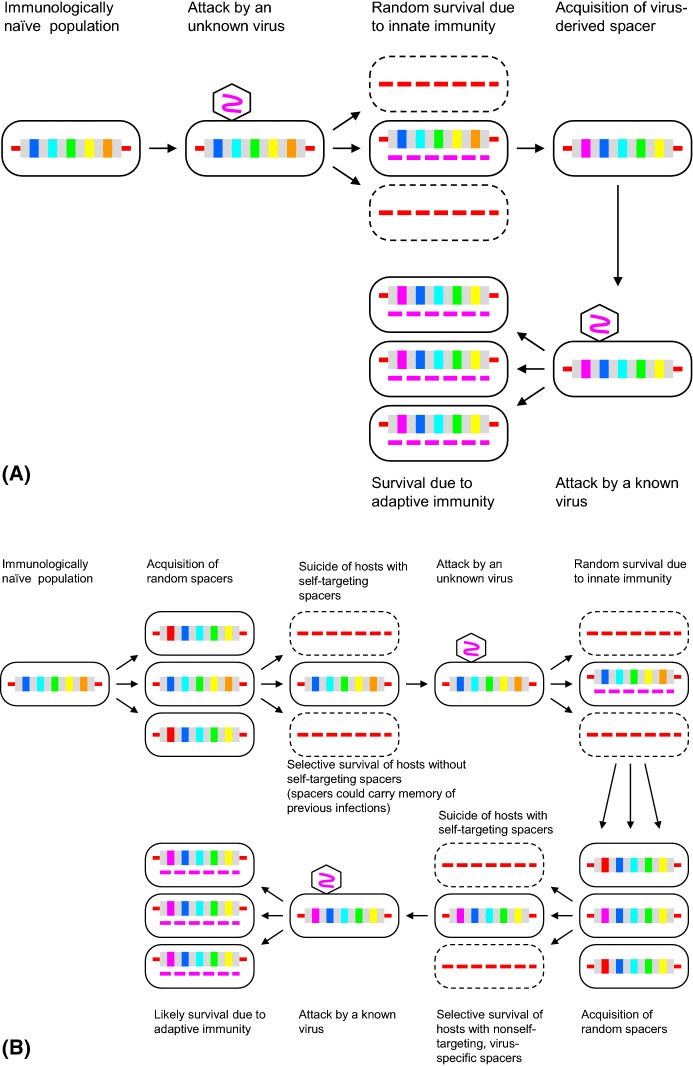
The Lamarckian and Darwinian modalities of CRISPR-Cas. **a** Efficient self versus non-self discrimination: Lamarckian mechanism. **b** Limited self versus non-self discrimination: Darwinian mechanism. Adapted from Koonin and Wolf (2016) under Creative License

As soon as detailed, even if, at the time, speculative scheme of CRISPR-Cas function has been proposed, the idea presented itself that these systems of adaptive immunity function via a genuine Lamarckian mechanism, i.e. Inheritance of Acquired adaptive Characters (IAC) (Makarova et al. 2006). The IAC mechanism, as distilled in the spirit of Lamarck albeit in modern terms, has two essential aspects: (1) specific, heritable changes in the genome caused by an external factor, (2) specific phenotypic effect of those changes that constitutes adaptation to the causative factor. At face value at least, the CRISPR-mediated immune response involves both these components (Fig. 1a) (Koonin and Wolf 2009). First, an external factor, namely, a virus infection or invasion of another form of foreign DNA, such as a plasmid, results in a modification of a specific locus in the genome, namely a CRISPR array, of a kind that is unique to the given factor, i.e. incorporation of a piece of the invading DNA as a CRISPR spacer. Second, the inserted spacer is transcribed to produce a CRISPR-RNA that is employed as a guide to recognize and inactivate the cognate foreign DNA (Fig. 1). The highly specific adaptation to the external factor that caused the unique genomic alteration is apparent and undeniable.

The IAC, obviously, is a torturous subject in modern biology (Gissis and Jablonka 2011). Jean-Baptiste Lamarck was the first to propose a coherent account of biological evolution, and he perceived IAC to be the primary if not the only route of evolutionary change (Burkhardt 2013; Lamarck 1809). Charles Darwin emphasized random heritable changes as the principal source of variation (Darwin 1859) but in his later writings, particularly, in the last editions of the *Origin of Species*, increasingly invoked IAC as an important factor of evolution, apparently, because he held growing doubts about the sufficiency of random, small changes as the sole material for evolution (Darwin 1872). However, the subsequent developments in evolutionary biology, including numerous experiments, perhaps, most notably, the famous fluctuation test of Luria and Delbruck, have demonstrated the central role of random mutations in adaptation processes (Hershberg 2015; Luria and Delbruck 1943). Conversely, IAC had been discredited by experiments that aimed to test the plausibility of such a mechanism but came back essentially empty-handed, such as the notorious work of August Weissmann with rats’ tails (Droscher 2015), and more dramatically, by experiments that claimed confirmation of IAC, but turned out to be poorly reproducible and potentially fraudulent, like those of Kammerer with toads’ coloring, although reassessment of those results in terms of epigenetics might still be due (Vargas et al. 2017). Worse, IAC, or “Lamarckism” became eponymous with a variety of pseudo-scientific fads, the most damaging one being the infamous Lysenkoism (Soyfer 1994, 2001). Yet, over the last two decades or so, the discovery of pervasive, heritable epigenetic changes directly caused by environmental factors as well as various findings on apparent non-random, directional mutations have suggested partial rehabilitation of IAC (Gissis and Jablonka 2011). All this evidence notwithstanding, I submit that the characterization of the mechanism of CRISPR-Cas as an adaptive immunity system with genetic memory was the turning point for IAC (Koonin 2011; Koonin and Wolf 2009, 2016).

Phenomenologically, the CRISPR-mediated immunity is endowed with all the ingredients of IAC, or Lamarckian evolution: the genome of a bacterium or archaeon is modified in a highly specific manner, in response to a specific environmental challenge (such as virus infection), resulting in a highly specific and efficient adaptation to that particular challenge (Fig. 1) (Koonin 2011; Koonin and Wolf 2009, 2016). The realization of the apparent Lamarckian character of the CRISPR-mediated immunity stimulated examination of many other phenomena that involve seemingly non-random genomic changes from the perspective of IAC (Table 1). As a result, several processes, such as stress-induced mutagenesis and certain types of horizontal gene transfer, have been classified as “quasi-Lamarckian” (Koonin 2011; Koonin and Wolf 2009). Moreover, at least one branch of the eukaryotic RNAi network, the piRNA systems, clearly resembles CRISPR-Cas even though the molecular mechanisms and enzymatic machineries involved in the two processes are unrelated. The piRNA system employs transcripts of integrated copies of transposons to silence the related integrated elements by directing histone methylation. As in the case of CRISPR-Cas, this is a defense system with genomic memory, i.e. a (quasi)Lamarckian systems. Recently, a remarkable Lamarckian-type antivirus defense mechanism has been discovered in unicellular eukaryotes. This form of defense involves a small virus that integrates into the genome of the protist host, is activated by infection of a giant virus and protects the host from the latter (Fischer and Hackl 2016). The analogy with CRISPr-Cas is effectively complete despite the fact that the proteins and specific mechanisms involved are unrelated (Koonin and Krupovic 2016). Thus, the clearest examples of Lamarckian evolution appear to be adaptive defense systems with genomic memory which is not surprising because IAC, by definition, involves targeted genome modification.

**Table 1:** Lamarckian and quasi-Lamarckian phenomena

Phenomenon	Biological role/function	Phyletic spread	Lamarckian (IAC) criteria		
			Genomic changes caused by environmental factor	Changes specific to relevant genomic loci	Changes provide adaptation to the causative factor
*Bona fide Lamarckian (?)*					
CRISPR-Cas with strong self versus non-self discrimination (e.g. subtype I–E)	Defense against viruses and other mobile elements	Many archaea and bacteria	Yes	Yes	Yes
piRNA	Defense against transposable elements in germline	Animals	Yes	Yes	Yes
HGT (specific cases)	Adaptation to new environment, stress response, resistance	Archaea, bacteria, unicellular eukaryotes	Yes	Yes	Yes
Virophage-mediated defense against giant viruses in protists	Antivirus defense	Unicellular eukaryotes	Yes	Yes	Yes
*Quasi*-*Lamarckian*					
CRISPR-Cas with limited self versus non-self discrimination (e.g. subtype I!-A)	Defense against viruses and other mobile elements	Many bacteria	Yes	Partial	Yes
HGT (general case)	Diverse innovations	Archaea, bacteria, unicellular eukaryotes	Yes	No	Yes/no
Stress-induced mutagenesis	Stress response/resistance/adaptation to new conditions	Ubiquitous	Yes	No or partial	Yes (but general evolvability enhanced as well)

Whether or not a particular process qualifies as a bona fide case of IAC, or Lamarckian evolution, hinges on the specificity of the mutations involved. Traditionally, the concept of the Lamarckian mechanism of evolution is predicated on a high specificity of mutations, i.e. only the mutations that are adaptive with respect to the respective causative factor are supposed to occur. In the case of an adaptive immune system, such as CRISPR-Cas, this requirement boils down to the fidelity of self versus non-self discrimination. Several recent observations indicate that CRISPR-Cas systems differ from each other in that respect so that the specificity towards foreign target DNAs is at least in part determined by selection.

In CRISPR-Cas systems, self versus non-self discrimination occurs at two stages. First, discrimination obviously is essential during interference: the CRISPR machinery must not target the spacer itself within the repeat array. Such targeting would cause DNA damage and potentially, cell death. Most of the CRISPR-Cas systems avoid this outcome through the requirement for the PAM that is involved in both adaptation and inerference, and consists of a short sequence located next to the protospacer that is recognized by the adaptation complex and is essential for spacer acquisition (Deveau et al. 2008; Heler et al. 2015; Leenay et al. 2016; Mojica et al. 2009; Redding et al. 2015; Wang et al. 2015). Although the PAM is a short (typically, 2–3 base pairs), partially redundant sequence signature, it is strictly avoided in the CRISPR, thus preventing self-targeting (Westra et al. 2013). Type III CRISPR-Cas systems do not to require a PAM and instead apparently avoid self-targeting due to the requirement of non-complementarity between the crRNA and the target DNA in the sequence adjacent to the spacer, which appears to be an additional safeguard against self-destruction in all CRISPR-Cas systems (Marraffini and Sontheimer 2010).

The other discrimination step involves distinguishing between foreign and host DNA at the adaptation stage. Apart from the specific context of the CRISPR array, the PAM is effectively useless for self versus non-self discrimination because, whatever the information content of the motif, the host genome, being much larger than the genome of the targeted selfish element, will contain many more copies of the PAM. Increasing the size and specificity of the PAM and selecting the host for avoidance of the PAM sequence would reduce the effectiveness of CRISPR-Cas in defense because, should this be the case, many genomes of MGE, especially small ones, would contain no or too few copies of the PAM to allow efficient adaptation and protection. Apparently, the selection for effective defense outcompetes selection for avoidance of self-recognition because all so far identified PAMs are short and partially degenerate.

An obvious way to address the self versus non-self discrimination problem is to examine the spacer content of the CRISPR arrays. A recent comprehensive analysis has shown, in a general agreement with earlier, more anecdotal observations, that, although the fraction of spacers with perfect matches in the sequence databases was only about 7%, the majority of these hits came from viruses, and nearly all remaining ones could be traced to other MGE (Shmakov et al. 2017a, b). In other words, there is (virtually) no memory of autoimmunity in the CRISPR arrays. At face value, these observations could be interpreted as evidence of highly efficient discrimination. However, the crucial aspect of these findings is that they pertain primarily to spacers that have been fixed in the microbial population or at least have spread through thousands of cell divisions and hence have been subject to selection that could have eliminated self-targeting spacers. Indeed, recent unbiased analyses of spacer acquisition yield a more complex picture. In an assay for spacer acquisition by the type I-E CRISPR-Cas system of *Escherichia coli*, where the experimental setup prevented cell killing by self-targeting spacers, a substantial excess of spacers from plasmid DNA over those from chromosomal DNA was observed (Yosef et al. 2012). In contrast, experiments with the type II-A CRISPR-Cas system from *Streptococcus thermophilus* provide evidence of apparently random, indiscriminate spacer acquisition (Wei et al. 2015). When the nuclease activity of the endonuclease Cas9 was knocked out and the suicidal effect of autoimmunity was accordingly prevented, the overwhelming majority of the inserted spacers were found to originate from the host genome. The implication of this experiment is as startling as it is obvious: apparently, in this case, the CRISPR-Cas system is extremely wasteful, with the majority of cells committing suicide, so that upon an attack by a selfish element, the few that incorporate spacers homologous to the invader genome could survive (Fig. 1).

The molecular insights into the self versus non-self discrimination by CRISPR-Cas systems are limited but do point to some specific mechanisms. A breakthrough study on spacer acquisition by the *E. coli* type I-E CRISPR-Cas system has demonstrated a 100–1000 excess of foreign (plasmid) DNA over the host DNA among the inserted spacers and shown that spacer acquisition requires active replication of the protospacer-containing DNA, with spacers being acquired primarily at stalled replication forks (Levy et al. 2015). Therefore, small, fast replicating plasmid genomes are much more efficient as a source of spacers than the host DNA. These findings are compatible with earlier observations in the archaeon *Sulfolobus islandicus* indicating that acquisition of spacers from an infecting virus genome required its active replication (Erdmann et al. 2014). Detailed analysis of the spacer acquisition process by subtype I-E CRISPR-Cas system has mapped the regions of active spacer capture between a stalled replication fork and a Chi site (Wigley 2007), and shown that acquisition is substantially reduced in RecB,C,D mutants. Thus, in this system at least, spacers appear to be derived primarily from the products of RecBCD-catalyzed DNA degradation that are produced during the repair of double-stranded breaks associated with stalled replication forks. These experiments identify a mechanism of self versus non-self discrimination by the CRISPR-Cas machinery that is not based on any intrinsic differences between foreign and host DNA but rather on the much greater density of replication forks, and accordingly, of double-strand breaks in the former (Courcelle et al. 2015). This mechanism appears to involve an intimate connection between CRISPR-Cas immunity and DNA repair. In addition to the preference for actively replicating DNA, which results in preferential incorporation of spacers from MGE, some CRISPR-Cas systems (in particular, type III) require active transcription of the target such that the first step of interference is the cleavage of transcripts which is a pre-requisite for subsequent DNA cleavage.

Another mechanism of self versus non-self discrimination by subtype I-E and, apparently, at least some other CRISPR-Cas systems is the so-called priming whereby the acquisition of spacers from DNA containing at least one (partial) match a pre-existing spacer in the given host is strongly stimulated compared to the acquisition from DNA that lacks such spacer-matching sequences (Datsenko et al. 2012; Fineran et al. 2014; Savitskaya et al. 2013; Xue et al. 2015). Unlike unprimed acquisition, which depends only on Cas1 and Cas2, priming requires the involvement of the entire set of Cas proteins. Thus, it appears that, after recognizing a sequence related to the cognate protospacer, the Cas machinery efficiently generates new spacers, without dissociating from the target DNA and without a strict requirement for the PAM. This results in a strong enhancement of self versus non-self discrimination although the details of the mechanism remain to be elucidated. Apart from the replication-dependent discrimination and priming, at least some CRISPR-Cas systems are normally repressed and are induced only upon infection, thus further curtailing the deleterious effect of autoimmunity (Westra et al. 2015).

As follows from the above, there are currently more open questions than definitive answers on self versus non-self discrimination by CRISPR-Cas systems. Nevertheless, even the available data make it clear that variants of CRISPR-Cas differ in both the specific mechanisms and the efficiency of such discrimination. It appears that, in most if not all cases, there is no straightforward, highly efficient means for the recognition of foreign genetic material akin to that exercised by prokaryotic restriction-modification (RM) modules which “tag” host DNA by methylation, protecting it from cleavage (Pingoud et al. 2014). The mechanisms currently discovered for CRISPR-Cas, such as preferential use of actively replicating or actively transcribed DNA for adaptation, or priming, ensure only partial discrimination. Thus, the near perfect specificity for spacers originating from the mobilome that is observed in CRISPR arrays appears to result, primarily, from selection. In some of the CRISPR-Cas systems, CRISPR-Cas adaptation seems to involve extreme wastefulness whereby the number of cells that die due to autoimmunity exceeds that of cells surviving infection thanks to incorporation of antivirus spacers by orders of magnitude. These findings push the CRISPR-Cas systems into the domain of “quasi-Lamarckian” phenomena that combine directed mutations driven by environmental factors with selection [10, 11]. CRISPR-Cas appears to rely on a “semi-random” insertional mutagenesis where the insertion site is highly specific (restricted to the CRISPR array) but the inserted sequences (spacers) are chosen non-specifically or at best with an incomplete specificity (bias towards foreign genetic elements). At least in some CRISPR-Cas variants, most of the insertions come from the host (self) genome and are accordingly deleterious (often lethal) due to autoimmunity. Nevertheless, selection for resistance to virus infection that is provided by occasional beneficial mutations (insertions of spacers from viral or other MGE DNA) outweighs the damage from autoimmunity and is sufficient to maintain the CRISPR-Cas system throughout the evolution of nearly all archaea and many groups of bacteria (see the discussion of the conditions for CRISPR-Cas retention below). The key ingredient of the Lamarckian evolutionary process (IAC), namely, the direct induction of specific, adaptive mutations by the environmental challenge, appears to be manifested to different extents in different CRISPR-Cas systems. Selection among cells incorporating different spacers seems to be a major aspect of the CRISPR-mediated evolution of virus-resistant strains. Depending on the level of self versus non-self discrimination, these evolutionary processes can be thought of as spanning the continuum, from the classical Darwinian scheme whereby the mutational process is largely random (and hence wasteful), whereas specificity and adaptation are achieved via selection, to the bona fide Lamarckian scenario where mutations are precisely directed (Fig. 1). In a stark contrast, the type I-E CRISPR-Cas system seems to operate via a bona fide Lamarckian mechanism where the mutational process is dominated by directional, adaptive mutations, which is achieved via the coupling of spacer acquisition with replication accompanied by the DSB formation and the priming mechanism.

**Fig. 2:**
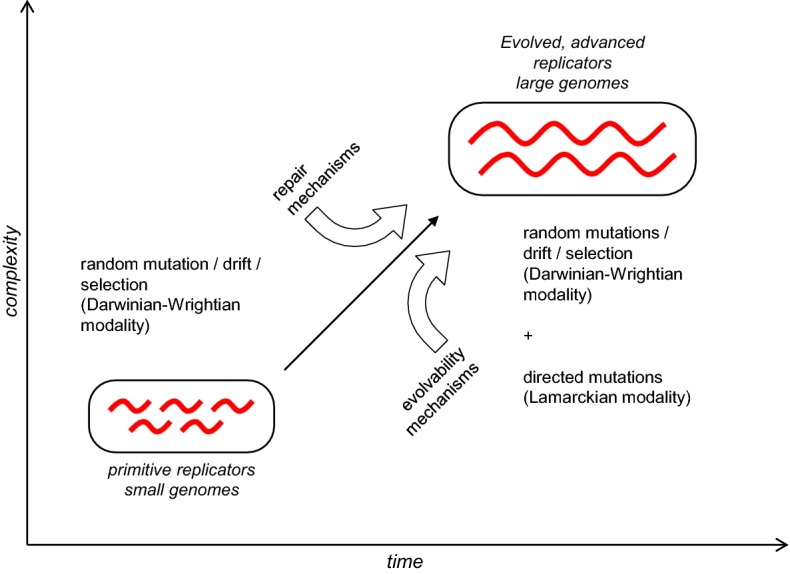
Evolution of genome repair systems, adaptive immunity and evolvability mechanisms. Adapted from Koonin and Wolf (2016) under Creative License

Despite many remaining uncertainties, the current findings on the interplay between selection and directed mutation in CRISPR-Cas response convey an important conceptual message by showing that, in real life, different modes of evolution hardly exist in pure forms but rather blend in different proportions. With regard to general aspects of evolution, CRISPR-Cas systems perfectly illustrate another key point, namely the fundamental difference between the Darwinian (selection) and Wrightian (genetic drift) modes of evolution, on the one hand, and the Lamarckian mode, on the other hand. Darwinian evolution that is based on negative and positive selection acting on random mutations as well as genetic drift (Wrightian evolution) are intrinsic features of replicator systems which are inherently error-prone. These mechanisms have been operating since the origin of the first replicators which can be considered equivalent to the origin of life (Koonin 2011). In contrast, Lamarckian evolution requires elaborate machinery for “natural genome engineering”, such as the CRISPR-Cas systems. The advent of increasingly complex life forms was enabled by increasing replication fidelity through the evolution of DNA repair mechanisms (Penny 2005; Wolf and Koonin 2007). The evolvability mechanisms underlying the (quasi)Lamarckian evolution seem to have evolved jointly with and/or from repair processes (Koonin and Wolf 2016) (Fig. 2). The two classes of mechanisms are tightly linked, both functionally and evolutionarily. The CRISPR adaptation stage includes repair of the gaps in the DNA that are generated during spacer insertion. Furthermore, self versus non-self discrimination in at least some CRISPR-Cas systems relies on repair processes as discussed above (Levy et al. 2015). Moreover, there are some indications that CRISPR-Cas systems could contribute to repair, in particular, that knockout of the *E. coli cas1* gene leads to deficiencies in various forms of repair (Babu et al. 2011). As a historical aside, the first detailed analysis of the Cas protein sequences and predicted functions has led to the hypothesis that these proteins together comprised a novel repair system (Makarova et al. 2002). Although this prediction missed the mark, not recognizing the defense function of CRISPR-Cas, there have been good reasons to infer a repair function because the repertoires of proteins that are involved in repair and in CRISPR immunity (primarily, various nucleases and helicases) clearly overlap. Strong connections to repair also exist for other evolvability mechanisms that involve (quasi)Lamarckian phenomena (Table 1), such as stress-induced mutagenesis and HGT. Indeed, it is hard to imagine how (quasi)Lamarckian mechanisms could be implemented without the close involvement of repair mechanisms because the formation of genomic memory of environmental cues, which is at the core of IAC, necessarily requires efficient repair of the genomic DNA.

### The conditions for genomic memory persistence

The CRISPR-Cas systems endow prokaryotes with highly specific and efficient defense against viruses and other parasitic genetic elements. Nevertheless, while these systems are nearly ubiquitous among archaea, they show patchy distribution among bacteria, being represented in only about 30% of the currently sequenced bacterial genomes (Makarova et al. 2015). Evolutionary reconstructions indicate that CRISPR-Cas loci are frequently lost during bacterial evolution (Puigbo et al. 2017). What are the causes of such non-uniform distribution and highly dynamic evolution of prokaryotic adaptive immunity? The frequent loss of CRISPR-Cas loci implies that this system is costly, and indeed, the sources of the cost appear clear. The first one is autoimmunity that, as described above, could be a major burden in the case of at least some CRISPR-Cas systems that show inefficient self versus non-self discrimination. The other source of cost appears to be the impediment of horizontal gene transfer (HGT) caused by the activity of CRISPR-Cas (Bondy-Denomy and Davidson 2014; Marraffini and Sontheimer 2008; Samson et al. 2015; Weinberger and Gilmore 2012). The HGT is a major factor of evolution in prokaryotes and is thought to be essential for long term survival of microbial populations, as a counter-balance against accumulation of deleterious mutations (Muller’s ratchet), and for short term adaptation (Iranzo et al. 2016; Takeuchi et al. 2014).

**Fig. 3:**
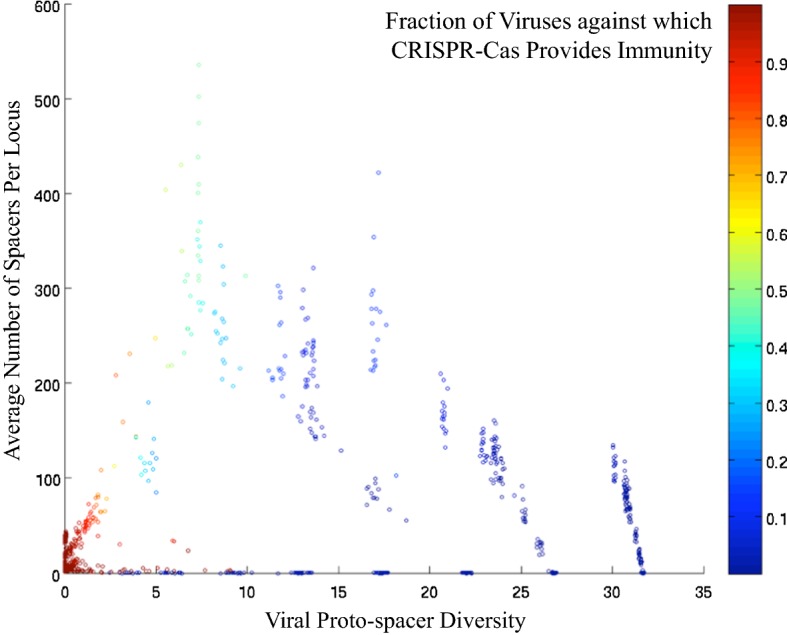
The non-monotonic dependency of the efficacy of CRISPR-Cas immune memory of parasite diversity. Adapted with permission from Weinberger et al. (2012)

Analysis of an agent-based mathematical model of the coevolution of parasites with hosts that possess both innate immunity and the more efficient but also more costly adaptive immunity (such as CRISPR-Cas) has shown a non-monotonic dependency of the fitness effect of adaptive immunity on parasite diversity (Fig. 3) (Weinberger et al. 2012). This cost–benefit analysis demonstrates that, at both low and high values of parasite diversity, the cost of maintaining adaptive immunity outweighs the benefit (heritable protection against the parasites), and accordingly, adaptive immunity is rapidly lost. At intermediate diversity, however, the benefit is maximized, and adaptive immunity is retained. Without going into the mathematical details, an intuitive interpretation of this non-monotonic curve does not appear difficult. At low parasite diversity, the cost of adaptive immunity is not worth paying because the less efficient but also less costly innate immunity is sufficient for resistance. Conversely, at extremely high parasite diversity, immune memory ceases to be beneficial because no parasite can be expected to be encountered more than once. A more detailed analysis of the mathematical model that included simulation of the growth of a CRISPR array suggests that the spacers accumulate with the increase of parasite diversity such that the maximum array length is reached immediately before the collapse and subsequent loss of adaptive immunity caused by an overwhelming diversity of the parasites (Fig. 3) (Weinberger et al. 2012). Recent experimental results on laboratory evolution of CRISPR-Cas appear to be compatible with these predictions (Morley et al. 2017; van Houte et al. 2016a, b). The link between environmental variation and the evolution of genomic or epigenomic memory suggested by this analysis is likely to be relevant beyond the domain of adaptive immunity.

### General implications of the (quasi)Lamarckian character of CRISPR

The description of the CRISPR-Cas, piRNA and some forms of HGT as (quasi)Lamarckian phenomena has been criticized, firstly, because this description seems valid only when the organismal level of selection is considered (Poole 2009) and secondly, because historically, Lamarckian evolution implies a teleological character of evolution (Weiss 2015). Both these criticisms indeed address major aspects of the evolutionary process but both appear to be readily answerable. As discussed above, the (quasi)Lamarckian phenomena are based on evolved mechanisms that could only emerge in relatively complex life forms, such as the first cells (Koonin and Wolf 2016). These mechanisms have nothing to do with teleology but rather emerged under the pressure to evolve efficient phenotype evolvability by biasing the mutational process and restricting mutations to specific genomic loci.

Evolution of evolvability has been the subject of a long-standing controversy (Kirschner and Gerhart 1998; Wagner 2017). However, detailed examination of putative evolvability mechanisms, such as CRISPR-Cas, piRNA and some other phenomena, including microbial gene transfer agents, leave little doubt that these cellular systems have evolved under pressure for introducing specific types of heritable changes into the genomes (Koonin 2011). Put somewhat boldly, but I think appropriately, these are dedicated devices for genome evolution. It is crucial to emphasize that the emerging concept of the role of IAC in organismal evolution is fully founded on distinct, elaborate molecular systems that do not involve any new elementary mechanisms. The familiar molecular biology and biochemistry account for all these processes but the combination of the elementary mechanisms can be unusual, and the emergent phenomena are the “Lamarckian-type” routes of evolution. This new understanding has nothing in common with Lamarck’s favorite idea of innate tendency for perfection driving biological evolution let alone with the Lysenkoist quackery.

## Matters of life and death: coupling immunity with programmed cell death or dormancy induction in prokaryotes

Genetic parasites (MGE) are inherent to replicator systems (Forterre and Prangishvili 2013; Koonin and Dolja 2013; Koonin and Starokadomskyy 2016). As demonstrated by both theoretical analysis and empirical data, virtually no cellular life form can eliminate parasitic genetic elements (Iranzo et al. 2016; Smith 1979; Szathmary and Maynard Smith 1997), and most organisms host diverse classes of such elements including viruses, transposons and plasmids (Koonin and Dolja 2014). Thus, the entire history of life is a story of incessant arms races between parasites and hosts during which both sides evolve diverse offence, defense, and counter-defense strategies (Forterre and Prangishvili 2009, 2013; Koonin and Dolja 2013). Nearly all cellular life forms, with the exception of some intracellular parasitic bacteria, possess multiple anti-parasite defense mechanisms that function on different principles (Koonin et al. 2017a, b; Makarova et al. 2013a, b). The major defense strategies include: (1) resistance, when receptor for a particular parasite, such as a virus, is lost or mutates to a form that precludes the entry of the parasite into the host cell, (2) innate immunity, i.e. diverse mechanisms that actively prevent the reproduction of a broad range of parasites, (3) adaptive (acquired) immunity, i.e. mechanisms that collect information on a specific parasite and utilize it to abrogate the reproduction of that parasite, and iv) programmed cell death (PCD) (and possibly more broadly, programmed suicide of an organism) when an infected cell instigates a self-destruction program that prevents parasite reproduction from reaching completion and thus protects other cells from infection (Makarova et al. 2013a, b; Netea et al. 2011; Rimer et al. 2014). In bacteria, the functional systems that cause PCD, in many cases, actually induce dormancy (stasis), i.e. a non-reproducing cellular state characterized by extremely low metabolic activity (Kint et al. 2012; Lewis 2007, 2010); hereinafter, I generically refer to PCD when discussing mechanisms inducing either dormancy or cell death. The PCD can be considered a form of innate immunity inasmuch as the suicidal response is triggered indiscriminately by different pathogens. Nevertheless, given the fundamental biological difference between immunity responses, in which cellular organisms incapacitate pathogens, and PCD, whereby cells kill themselves, in a display of “altruism”, I henceforth treat these strategies as distinct.

The CRISPR-Cas systems showcase tight, intricate connections between immunity and PCD (Koonin and Makarova 2013; Makarova et al. 2012). Even apart from PCD, a dedicated machinery for altruistic self-destruction, immunity mechanisms carry an inherent threat of suicide (Koonin 2017; Koonin and Zhang 2017). Immunity is a collection of mechanisms for abrogation of reproduction and destruction of parasites, above all, MGE including viruses. Given the fundamental unity of the genetic systems across all life, cell or virus, immunity is dangerous by design because immune systems will inevitably attack the host itself unless kept in check via dedicated self versus non-self discrimination mechanisms. In most general terms, this is a consequence of the laws of thermodynamics that prohibit error-free information transmission without commensurate energy expenditure (Koonin 2016; Shannon and Weaver 1963). The numerous, often devastating human autoimmune diseases are an obvious case in point (Bach 2003; Kronenberg 1991). As discussed above, autoimmunity has been demonstrated for the CRISPR-Cas systems (Hooton and Connerton 2014; Sorek et al. 2013; Stern et al. 2010), in accord with the notion that it is intrinsic to immunity. Moreover, at least some CRISPR-Cas variants appear to insert primarily spacers from the host genome (Wei et al. 2015) although only those few that incorporate parasite-specific spacers survive (Shmakov et al. 2017a, b). Such strong selection for cognate spacers is possible only when the benefit of the protection from parasites is substantial, and/or when the immune systems themselves possess properties of selfish elements and become “addictive” to the host (see discussion below).

Notably, apart from the suicidal potential of immune systems, the respective genomic loci often also include dedicated PCD modules, such as toxins-antitoxins (TA), and on other occasions, some proteins are shared by the immune and PCD systems. Several such connections with PCD are present in CRISPR-Cas systems (Makarova et al. 2012). One of the key proteins involved in the first, adaptation phase of the CRISPR response, Cas2, is a homolog of the VapD family of mRNA interferases, which are toxins that cause dormancy by cleaving mRNA molecules inside the ribosome (Makarova et al. 2006; Makarova et al. 2011a, b). Indeed, non-sequence-specific nuclease activity of several Cas2 proteins against both DNA and RNA, but typically, with a preference for RNA substrates, has been demonstrated (Beloglazova et al. 2008; Dixit et al. 2016; Gunderson et al. 2015; Ka et al. 2014; Nam et al. 2012). The primary role of Cas2 in CRISPR-Cas is that of a structural scaffold of the adaptation complex in which the active endonuclease (integrase) component is Cas1 (Amitai and Sorek 2016; Nunez et al. 2014, 2015). The interferase catalytic site is conserved in many but not all Cas2 proteins, and is not required for adaptation (Nunez et al. 2014). Thus, at least in certain CRISPR-Cas systems, Cas2 might play a secondary role as a RNase, possibly, in the capacity of a toxin (Makarova et al. 2012), although catalytically active Cas2 proteins do not appear to be toxic when overexpressed in *E. coli*.

Many CRISPR-Cas systems, especially, those of type III, also encompass additional nucleases, in particular, (predicted) RNases of the HEPN (Higher Eukaryotes and Prokaryotes Nucleotide-binding domain) superfamily (Anantharaman et al. 2013; Makarova et al. 2014). The RNase activity of two of these proteins, Csm6 and Csx1, has been experimentally demonstrated (Jiang et al. 2016; Niewoehner and Jinek 2016; Sheppard et al. 2016). Most of the HEPN-containing Cas proteins additionally contain the CARF domain which adopts the Rossmann fold and is predicted to bind ligands, most likely nucleotides, and perform signaling functions (Makarova et al. 2014). Recently, the HEPN domain of the Csm6 protein of subtype III-A from *S. thermophilus* has been shown to cleave viral mRNAs after being activated by olioadenylates that are synthesized by the Cas10 proteins in response to target recognition and are bound by the CARF domain of Csm6 (Kazlauskiene et al. 2017; Koonin and Makarova 2017, 2018; Niewoehner et al. 2017). In this case, mRNA cleavage is a pre-requisite for viral genomic DNA cleavage and does not appear to represent toxic action. However, the Csm6 protein of the archaeon *Pyrococcus furiosus* that also consists of a CARF and HEPN domains is not required for the type III-B CRISPR-Cas interference (Elmore et al. 2016) suggestive of a different, accessory function for this protein. The HEPN domain superfamily consists of extremely diverse (predicted) RNases that are primarily involved in various defense functions. In particular, a highly abundant class of TA modules encompasses HEPN domain-containing proteins as the toxin moieties (Anantharaman et al. 2013). The HEPN domain-containing systems remain poorly functionally characterized but are common in prokaryotes, and are the most abundant TA variety in archaea (Anantharaman et al. 2013; Makarova et al. 2009). Accordingly, it appears likely that at least some of the HEPN domain-containing Cas proteins also possess toxin activity that could be activated allosterically through the CARF domain. In some CRISPR-Cas variants, the CARF domain is fused to predicted nucleases that are unrelated to HEPN, in particular, Cas4 homologs which adopt the Restriction Endonuclease fold (Makarova et al. 2014). This apparent interchangeability of CARF-linked nucleases suggests the intriguing possibility that many if not all of them can function as toxins that are regulated through ligand-binding by the respective CARF domains.

A CRISPR-associated toxin activity has been experimentally demonstrated for the Csa5 protein of the type I-A CRISPR-Cas system of the archaeon *Sulofolobus solfataricus*. Infection of *S. solfataricus* with the SIRV2 virus induced the expression of Csa5 to the toxic level and resulted in cell death, suggesting that the toxicity of this protein indeed represents a PCD response to virus infection (He et al. 2014). The Csa5 protein is the small α-helical subunit of the CRISPR RNA-processing complex of type I-A (Cascadelike complex) and does not appear to possess any nuclease activity (Daume et al. 2014), so the mechanism of toxicity remains obscure. These findings suggest that the CRISPR-associated toxicity is a broad phenomenon that goes beyond the known activities of toxic nucleases.

**Fig. 4:**
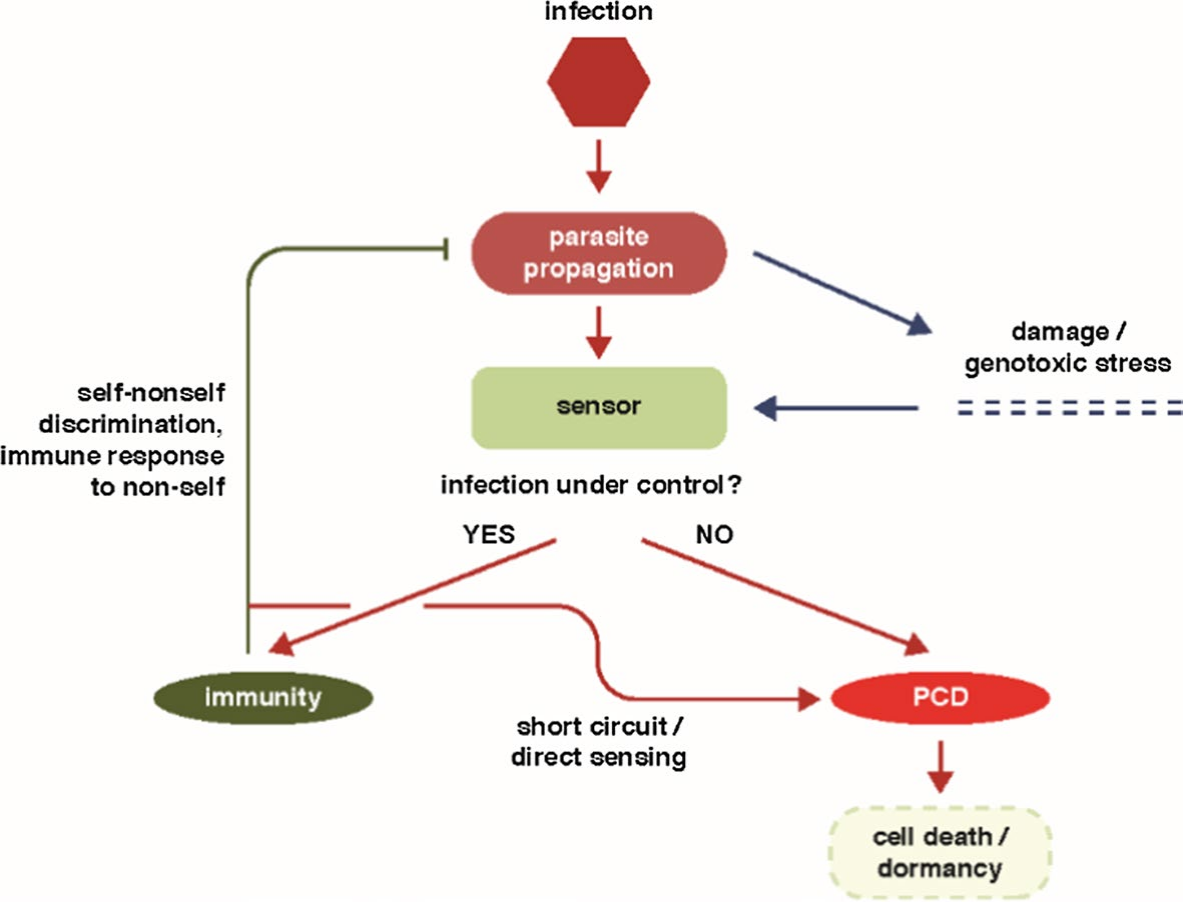
Coupling of immune response and programmed cell death/dormancy via stress sensors [JOURNAL]. Adapted with permission from Koonin et al. (2017a, b)

The recent discovery of new Class 2 CRISPR-Cas systems by a comprehensive search for genomic loci that encode large proteins containing putative nuclease domains that could function as CRISPR-Cas effectors, has revealed the most direct currently known link between CRISPR-Cas and PCD (Abudayyeh et al. 2016; Shmakov et al. 2015; Shmakov et al. 2017a, b; Smargon et al. 2017). Unlike all previously characterized members of the HEPN domain superfamily, the type VI effector proteins (Cas13) contain two HEPN domains that are both active RNases and are required for interference (Abudayyeh et al. 2016; Shmakov et al. 2015, 2017a, b; Smargon et al. 2017). In addition, Cas13a showed a distinct capacity that, although apparently highly unusual, in retrospect, could perhaps have been predicted. When primed with a cognate RNA, this protein becomes a promiscuous RNase that cleaves any RNA molecules present in the reaction mix with little sequence specificity (Fig. 4). A decrease in bacterial viability has been observed when Cas13a was coexpressed with the cognate RNA, suggesting dormancy induction (Abudayyeh et al. 2016). Given the apparent minor role of RNA bacteriophages in the bacterial virosphere (Koonin et al. 2015), it appears most likely that the principal functionality of type VI CRISPR-Cas is defense against DNA phages that is realized through the toxic effect that is triggered by the recognition of a cognate phage transcript and leads to dormancy or PCD.

Taken together, these observations on CRISPR-Cas along with those on other defense mechanisms, in particular, the thoroughly studied bacterial anti-phage defense system that centers on HEPN domain-containing RNases cleaving tRNAs (Kaufmann 2000; Klaiman and Kaufmann 2011; Uzan 2009), have been interpreted in terms of functional coupling between immunity and PCD/dormancy (Makarova et al. 2012). Two versions of such coupling have been considered. In the first, arguably, the most obvious scenario, PCD is the strategy of last resort whereby the defense system senses the impending failure to stop virus reproduction in the given cell and accordingly switches to the suicidal mode, sacrificing the infected cell but saving other cells in the population. Alternatively and perhaps less realistically, it has been speculated that, faced with rampant virus reproduction, the immune system would turn on the dormancy induction machinery, thus not only protecting the surrounding cells but potentially, giving the infected cell a chance to recover once the virus clears. The two strategies might not be completely distinct given that there is never a guarantee that a cell re-emerges from dormancy. The presence, in numerous CRISPR-*cas* loci, of genes encoding proteins, in which CARF domains are fused with diverse nucleases (Makarova et al. 2014), suggests that the CARF domain functions as a sensor of defeat of the immune system in the battle with the virus, probably, responding to alarmones that remain to be identified (Fig. 4). So far, this type of allosteric stimulation of the HEPN RNase activity has been demonstrated in the form of the oligoA-dependent pathway that triggers the immune response (Kazlauskiene et al. 2017; Koonin et al. 2017a, b; Niewoehner et al. 2017), but it appears likely that many variations on this theme exist, some of which trigger PCD.

What governs life-or-death decisions and why do organisms “bother” to evolve dedicated suicide machinery? Whether the cell that turns on the self-afflicting program kills itself right away or goes into dormancy, with a chance of comeback, the same factors determine the decision: the cell must “predict” the outcome of infection and act accordingly (Fig. 4) (Koonin et al. 2017a, b). If, after the immune system recognizes an invasion, the sensor module “predicts” that the onslaught is likely to be manageable, the immune system is mobilized to its full capacity. If, on the contrary, the forecast is dire, the self-destruction program is turned on. The signals read by the sensor are likely to differ among defense systems. In some cases, the cell damage (genotoxic stress) could be measured directly as illustrated by the tRNA-cleaving phage-defense pathway where different components sense double-stranded DNA breaks or dTTP accumulating during phage infection (Klaiman and Kaufmann 2011; Klaiman et al. 2014; Krutkina et al. 2016). The CARF domain, possibly, along with other ligand-binding domains, such as WYL (Makarova et al. 2014), is likely to be a toggle between adaptive immunity and PCD. Type VI CRISPR-Cas systems seem to short-circuit the typical defense relay by skipping or at least simplifying the damage-sensing step and having the main immune effector double as the suicide effector (Fig. 4). Indeed, the Cas13 effector proteins switch to promiscuous RNA cleavage in vitro where the only signal comes from the recognition of the target (Abudayyeh et al. 2016; Smargon et al. 2017). Type VI systems are rare among bacteria compared to types I, II and III (Shmakov et al. 2017a, b) which might reflect the high cost of these systems to the host due to their “panic” response to invading DNA. Nevertheless, sensing the target RNA concentration, yielding information on the multiplicity of infection and/or expression level of the virus genome, by the Cas13 proteins themselves, could occur even in this case. Conceivably, the more complex defense strategies that involve the dedicated sensor module (Fig. 4), such as Class 1 CRISPR-Cas, are beneficial under a wider range of conditions than simple ones, such as type VI CRISPR-Cas, which activate the self-destruction program at the first alarm.

Both immune systems with their suicidal proclivity, and especially, dedicated suicide devices are prone to misfiring and are thus costly for the organism. What are, then, the factors that determine the broad (although not universal) persistence of both these types of costly defense strategies? Mathematical modeling of the coevolution of different defense mechanisms with pathogens considered in the context of the biological features of defense systems seem to offer some clues (Koonin and Wolf 2015; Kumar et al. 2015). Detailed analysis of the coevolution models shows that, assuming some basal level of innate immunity, adaptive immunity and suicide can coexist only within a limited region of the parameter space where the efficacies of both types of defense are limited (Iranzo et al. 2015). Such a situation would correspond to the sensing toggle circuit, where the sensor “predicts” the outcome of infection and whether the immune system is likely to cope successfully (Fig. 4). These considerations on coevolution of the immune and suicidal defense strategies apply to both adaptive immunity, such as CRISPR-Cas, which is central to the response of organisms to familiar pathogens, and innate immunity, which acts against newcomers.

Furthermore, immunity-suicide coupling is favored when the defense circuitry contains dual function components that are involved both in immune and in suicidal activities (Iranzo et al. 2015). The CRISPR-Cas systems are particularly notable in this respect given that multiple essential as well as accessory Cas proteins, including Cas2, Csm6, Cas13 and others, appear to have evolved from toxins and, in addition to their exapted functions in CRISPR-Cas systems, might also switch to their toxic capacity when the suicidal program is launched.

## Guns for hire: evolutionary connections between CRISPR-Cas systems and mobile genetic elements

**Fig. 5:**
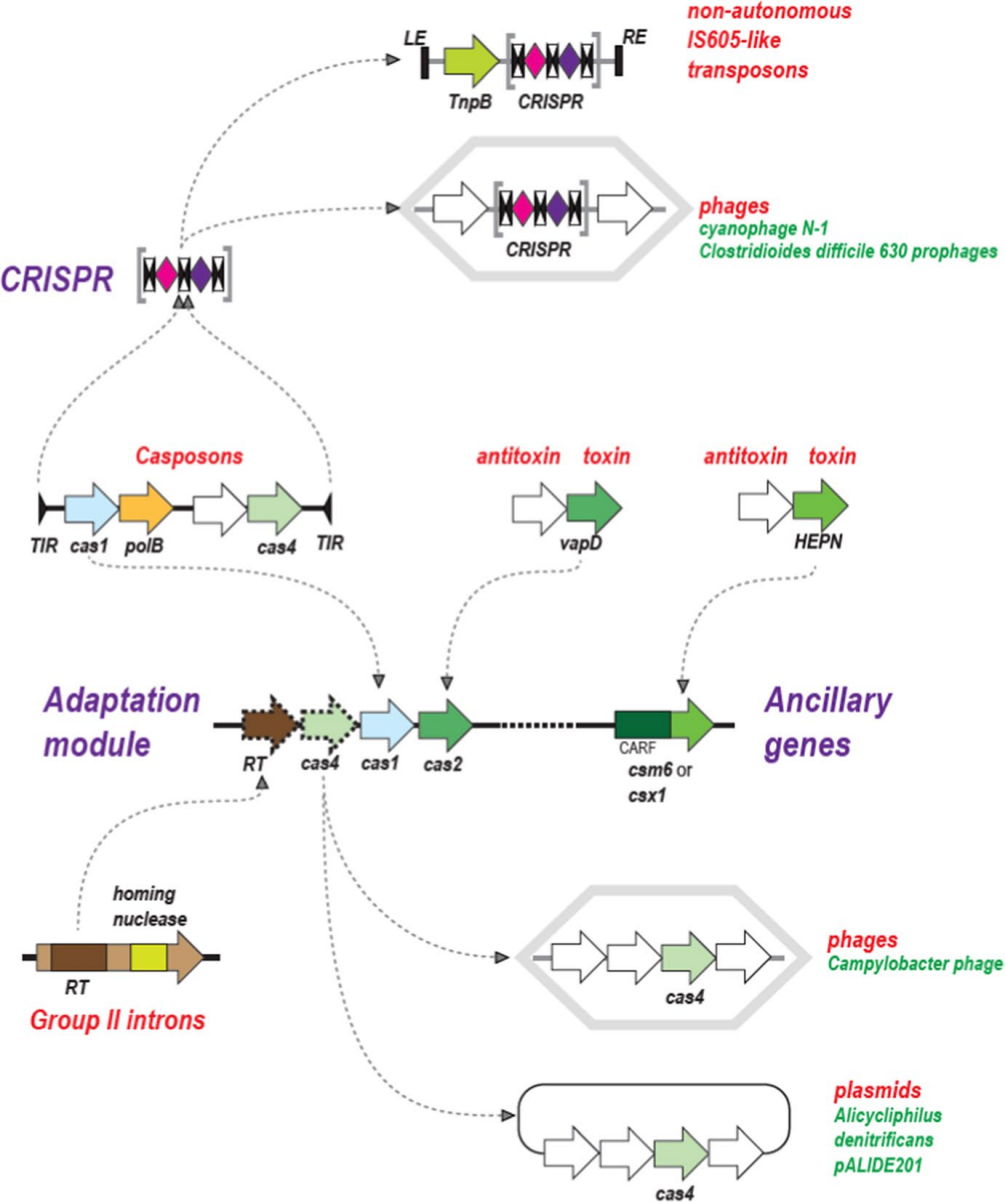
Evolutionary connections between mobile genetic elements and CRISPR-Cas systems. The arrows show putative ancestor-descendent relationships. Adapted with permission from Koonin et al. (2017a, b)

The third aspect of the emerging picture of CRISPR-Cas evolution that has major general implications for our understanding of evolution involves the multiple contributions of MGE to the origins of the prokaryotic adaptive immunity and the converse recruitment of defense systems or their components for antidefense functions by MGE (Koonin and Makarova 2017, 2018) (Fig. 5). In particular, the adaptation modules of CRISPR-Cas systems or at least the key enzyme involved in adaptation, Cas1, derive from a distinct family of transposons that have been dubbed casposons, to emphasize the fact they encode a transposase homologous to Cas1. The microbial adaptive immunity systems are thought to have evolved through a chance casposon insertion next to an ancestral innate immunity locus followed by immobilization of the casposon and loss of some of its genes. The repeats themselves might have originated from the duplicated target site of the casposon. Apart from the adaptation module, nucleases encoded by unrelated class transposons (TnpB proteins of IS605 superfamily transposons) gave rise to the effector nucleases of type II and type V CRISPR-Cas systems (Cas9 and Cas12, respectively). Notably, phylogenetic analysis clearly shows that effectors of different subtypes of type V evolved indendently from different TnpB subfamilies. The effectors of the RNA-targeting type VI (Cas13) evolved from yet a different class of MGE, namely, toxin-antitoxin modules which donated the HEPN domains, the RNase moieties of both microbial toxins and Cas13. Finally, the RT of the type III adaptation modules is a derivative of the RT of Group II introns, prokaryotic retroelements (Fig. 5).

Complementary to the multiple contributions of MGE to the evolution of CRISPR-Cas systems, substantial reverse gene flow, from CRISPR-Cas systems to MGE, has been discovered as well (Fig. 5). Specifically, minimal forms of type I CRISPR-Cas systems that apparently lack the interference capacity are present in a large family of Tn7 transposons, whereas type IV systems that, too, lack the interference module are carried by diverse plasmids. The roles of the CRISPR-Cas systems carried by transposons and plasmids remain to be elucidated, one intriguing possibility being that these systems mediated RNA-guided transposition. Additionally, several bacteriophages encode fully competent CRISPR-Cas systems that function against the host defense systems Finally, on multiple occasions, MGE recruit individual *cas* genes that either interact with the host CRISPR-Cas or are exapted for unrelated functions.

The multiplicity and diversity of exchanges between microbial immune systems and MGE clearly indicates that the connection is not fortuitous but rather reflects a deep evolutionary unity that is not limited to CRISPR-Cas but involves the entirety of defense mechanisms. Indeed, simple defense systems in prokaryotes, such as TA and RM modules themselves possess properties of MGE (Furuta and Kobayashi 2011; Kobayashi 2001; Van Melderen and Saavedra De Bast 2009; Van Melderen 2010). A more complex interplay between parasitism and defense is captured in the “guns for hire” concept, whereby homologous proteins, such as endonucleases, are utilized as offensive and defensive ‘weapons’, by MGE and defense systems, respectively (Koonin and Krupovic 2015a, b). Recruitment of transposons or their components apparently was central not only to the evolution of CRISPR-Cas but also to the origin of adaptive immunity in vertebrates (Kapitonov and Jurka 2005, Kapitonov and Koonin 2015, Koonin and Krupovic 2015a, b), the system of DNA elimination and rearrangement in ciliates (Allen and Nowacki 2017; Betermier and Duharcourt 2014; Dubois et al. 2012; Nowacki et al. 2011), and the piRNA machinery of germ line defense in animals (Aravin et al. 2007). Strikingly, a recently discovered mechanism of adaptive immunity against giant viruses of unicellular eukaryotes follows the same principle where small viruses, known as virophages, integrate into the host genome and protect the host against giant virus infection (Fischer and Hackl 2016; Koonin and Krupovic 2016). In each of these cases, integrases from unrelated transposons have been recruited for integration of genetic material and/or genome rearrangement that are central to the respective processes. A broad generalization seems to be in order: all molecular systems, many but not all of them with defense functions, that are involved in various forms of genome manipulation are evolutionarily linked to MGE, the quintessential genome editing molecular machines. Elucidation of the diversity and the intricacies of the interactions between MGE and cellular genome manipulation machineries, and development of a general theory of their coevolution are research directions for decades to come.

## Fundamental discoveries and major applications: two sides of the same coin

In the second decade of the twenty-first century, despite the unprecedented success in many research directions, funding for fundamental research is becoming increasingly problematic. With few exceptions, to get funded, research has to be “translational” or “applied”. The “CRISPR craze” is arguably one of the two or three most successful translational research stories of the century. Indeed, in short 4 years, the CRISPR technology has progressed from the discovery of a molecular mechanism to a suite of multimillion dollar applications that have become ubiquitous in laboratories and are making rapid strides into diagnostics and, eventually, clinic. Yet, one could argue that the story of CRISPR is primarily about research into fundamental biological mechanisms. Indeed, the CRISPR-Cas system has been discovered through a series of serendipitous findings in comparative genomics, and the intense research into its function started after its role in adaptive immunity has been first predicted from those comparative genomic clues and then demonstrated experimentally (Barrangou and Horvath 2017).

The CRISPR mechanism is highly non-trivial, and in my view, the study of these defense systems informs our understanding of biology at a high level of generality, bringing up issues of philosophical interest (Box 1). Prior to this discovery, natural genome engineering at this level of precision or mechanisms that would so closely match the definition of Lamarckian inheritance (IAC) have not been known. As discussed in this article, these features of CRISPR stimulated reassessment of many other genetic mechanisms that seem to show some “Lamarckian” features as well (Table 1). Two additional general phenomena have become apparent thanks to CRISPR: coupling between immunity and PCD, and the evolutionary entanglement between adaptive immunity and MGE. The latter trend testifies to fundamental principles of genome manipulation that are recapitulated in convergent evolutionary trajectories of diverse immune systems. As difficult as it is, in general, to infer the directionality of evolution in biology, the primacy of the MGE in this evolutionary interplay appears undeniable. The emergence and persistence of MGE is an intrinsic feature of replicator systems, and some of the common MGE have very simple organization, often with the integrase as the only gene. Clearly, the MGE provided the building blocks for elaborate genome manipulation machineries, such as adaptive immunity systems.

**Box 1:** The key general messages from CRISPR-Cas research

CRISPR-Cas systems appear to realize the Lamarckian evolutionary scenario
CRISPR-mediated immunity is apparently coupled to “altruistic” programmed cell death: cells “decide” to commit suicide when defense fails
Like all defense systems, CRISPR-Cas is costly, due to autoimmunity and curtailment of horizontal gene transfer, hence frequent loss of CRISPR-*cas* loci during evolution
At least some key components of CRISPR-Cas systems evolved from genes of mobile genetic elements which demonstrates tight coevolution of biological offense and defense
The essential biological feature of CRISPR-Cas—the ability to recognize and cleave unique genomic sites—makes them ideal genome engineering tools

A comparison of the mechanistic features of the prokaryotic adaptive immune systems, CRISPR-Cas, and the much more familiar vertebrate adaptive immunity could be instructive. At first glance, the two systems have little in common. In prokaryotes, adaptive immunity functions on the basis of nucleic acid complementarity and, in that respect, presents a closer parallel to the eukaryotic RNAi network (see above). In contrast, the vertebrate adaptive immunity is based on protein–protein (or less commonly, protein-nucleic acid or protein-carbohydrate) recognition. Furthermore, in contrast to the transgenerational inheritance of immunity in prokaryotes, the immune memory in vertebrates is limited to a single generation life span because immunological adaption occurs in somatic cells. Also, in contrast to the Lamarckian mode of evolution that is engendered by CRISPR-Cas systems (see above), vertebrate immunity follows a Darwinian scenario whereby the infectious agent selects from the pre-existing immunoglobulin diversity. However, apart from these differences, a profound commonality between the prokaryotic and animal versions of adaptive immunity is that adaptation in both cases occurs via genome rearrangement, and the two systems, have recruited unrelated transposases that mediate the respective rearrangements. Interestingly, it has been proposed that the numerous viral sequences integrated in animal genomes might serve as a reservoir of immunological memory (Hurwitz et al. 2017). However, concrete data in support of such a mechanism are presently lacking.

It is worth emphasizing that the same features that make CRISPR a powerful immune mechanism, namely, its ability to recognize and cleave unique DNA or RNA sequences with extremely high specificity and efficiency, make it so outstanding as genome editing tool. Put another way, CRISPR-Cas actually is a naturally evolved genome editing toolkit. Better yet, this toolkit has diversified through the course of the host-parasite coevolution, and functionally diverse CRISPR-Cas variants already have been harnessed for the respective, most suitable applications. The best case in point could be type VI, with the dedicated RNA-targeting effector Cas13, that has been rapidly adopted for RNA modification and detection with a single molecule sensitivity (Abudayyeh et al. 2017; Cox et al. 2017; East-Seletsky et al. 2017; Gootenberg et al. 2017; Murugan et al. 2017). Furthermore, a whole new family of applications has been developed by decoupling the recognition and the cleavage of the target as implemented in the “dead” variants of Cas9 or Cas13, in which the nuclease catalytic sites have been mutated.

The CRISPR-Cas case certainly is not unique when it comes to the utility of naturally evolved defense systems as molecular tools. The previous generation of genome editing methods that developed in the 1970s–1980s centered around the bacterial RM systems that are involved in innate immunity and show limited specificity towards the DNA sequences they recognize and cleave (Pingoud et al. 2014). Essentially by definition, innate immunity cannot match the specificity of adaptive immune mechanisms, but this shortcoming is partially compensated by the enormous diversity of the restriction endonucleases that has been successfully employed to support genome engineering throughout the first two decades of the genomic era, prior to the advent of CRISPR-Cas, and remain indispensable for many applications (Roberts et al. 2003, 2007). Coming back to adaptive immunity, animal antibodies, the key component of adaptive immunity, have been for decades providing essential methodology for the recognition of protein molecules in all areas of life sciences. Under a broader perspective, it stands to reason that any defense systems as well as other cellular systems that are based on molecular recognition have the potential to become biochemical tools. The advances of genomics and metagenomics show that we are hardly aware of all or even the majority of such systems that exist in nature, particularly, in the microbial world. As potent as the CRISPR-Cas methodology is, there is no obvious reason to expect that it is the final achievement in genome editing and regulation technology. Beyond doubt, open-ended exploration of natural genome engineering mechanisms brings new possibilities. It remains to be seen whether these discoveries reveal fundamental new biology as it happened in the case of CRISPR-Cas.
